# Orientation selectivity properties for integrated affine quasi quadrature models of complex cells

**DOI:** 10.1371/journal.pone.0332139

**Published:** 2025-09-29

**Authors:** Tony Lindeberg

**Affiliations:** Computational Brain Science Lab, Division of Computational Science and Technology, KTH Royal Institute of Technology, Stockholm, Sweden; Universidad de Guadalajara, MEXICO

## Abstract

This paper presents an analysis of the orientation selectivity properties of idealized models of complex cells in terms of affine quasi quadrature measures, which combine the responses of idealized models of simple cells in terms of affine Gaussian derivatives by (i) pointwise squaring, (ii) summation of responses for different orders of spatial derivation and (iii) spatial integration. Specifically, this paper explores the consequences of assuming that the family of spatial receptive fields should be covariant under spatial affine transformations, thereby implying that the receptive fields ought to span a variability over the degree of elongation. We investigate the theoretical properties of three main ways of defining idealized models of complex cells and compare the predictions from these models to neurophysiologically obtained receptive field histograms over the resultant of biological orientation selectivity curves. It is shown that the extended modelling mechanisms lead to more uniform behaviour and a wider span over the values of the resultant that are covered, compared to an earlier presented idealized model of complex cells without spatial integration.

More generally, we propose to, based on the presented results: (i) include an explicit variability over the degree of elongation of the receptive fields in functional models of complex cells, and that (ii) the suggested methodology with comparisons to biological orientation selectivity curves and orientation selectivity histograms could be used as a new tool to evaluate other computational models of complex cells in relation to biological measurements.

## Introduction

To understand the functional properties of the visual system, it is essential to aim at bridging the gap between computational models on one side and neurophysiological measurements on the other side. Specifically, whenever possible, it is desirable to construct theoretically principled models, which could then lead to a deeper understanding of visual processing modules and also generate predictions for further biological experiments. Regarding visual perception, it is in particular important to develop a good understanding of the receptive fields^*^ (Hubel and Wiesel [1–4]) in the early layers of the visual hierarchy, which constitute the fundamental primitives that the higher layers in the visual hierarchy are based upon.

(See supplementary information “S1 File” for complementary explanations of the concepts marked by a star^*^ in this introduction.)

One area where it indeed seems to be possible to bridge the gap between neurophysiological recordings and principled theory concerns the normative theory for visual receptive fields in Lindeberg [5]. This theory has been developed from principled assumptions regarding symmetry properties of an idealized vision system, and leads to a canonical family of linear receptive fields in terms of spatial derivatives of affine Gaussian kernels. Interestingly, the shapes of these idealized receptive fields do rather well correspond to the qualitative shapes of simple cells recorded by DeAngelis et al. [6,7], Conway and Livingstone [8] and Johnson et al. [9]; see Figs 12–18 in Lindeberg [5] for comparisons between biological receptive fields and idealized models.

One of the components in this normative theory for visual receptive fields is the assumption that the family of receptive fields ought to be covariant^*^ under spatial affine transformations, so as to enable more robust processing of the image data as variations in the viewing conditions imply variabilities in the image data caused by natural image transformations (see Lindeberg [10–12]). Specifically, if a visual observer views the same object from different distances and viewing directions, then the local image patterns will be deformed by the varying parameters of the perspective transformations, which to first order can be approximated by local affine transformations. In the area of computer vision, it has specifically been shown that the property of covariance under spatial affine transformations, referred to as affine covariance, enables more accurate estimation of surface orientation, compared to using only isotropic receptive fields that do not support affine covariance (Lindeberg and Gårding [13]).

To implement affine covariance in a vision system corresponds to using receptive field shapes subject to different affine spatial transformations, and will thereby specifically imply that receptive fields ought to be present for different degrees of elongation^*^. In a companion work (Lindeberg [14]), we have indeed investigated the consistency of that affine covariant hypothesis with neurophysiological measurements of the resultant of orientation selectivity curves obtained by Goris et al. [15]. There, we showed that predictions of orientation selectivity curves and orientation selectivity histograms generated from idealized models of simple cells in terms of Gaussian derivatives are for idealized model of simple cells in reasonable agreement with biological orientation selectivity histograms. Thereby, those results for simple cells are consistent with an expansion of the receptive field shapes over the degree of elongation in the primary visual cortex of higher mammals.

The modelling of complex cells^*^ performed in Lindeberg [14] was, however, based on very much simplified models in terms of *pointwise* non-linear combinations of responses of simple cells, and thus not involving any spatial integration of the non-linear combinations over extended regions in the image domain. Additionally, the modelling of complex cells in Lindeberg [14] was based on input from simple cells up to only order two, and not involving simple cells up to order 4, which were shown to lead to better agreement between the predicted orientation selectivity histograms and the actual biological orientation selectivity histograms accumulated for simple cells by Goris et al. [15]. The subject of this paper is to investigate a set of extended models of complex cells, and to demonstrate that these models offer a potential to lead to better agreement with biological orientation selectivity histograms compared to previous work.

In this way, we will thus specifically demonstrate that the resulting modelling of complex cells is also consistent with an expansion of the receptive field shapes over the degree of elongation, and in this way consistent with the wider hypothesis about affine covariant visual receptive fields, previously proposed in Lindeberg [5,10,14]. Based on these results, to be presented below, we propose to include an explicit expansion over the degree of elongation of the receptive fields as an essential component when modelling the computational function of complex cells.

While the specific models of complex cells to be considered in this treatment will be highly idealized, in terms of generalized quadratic energy models of the output of idealized simple cells, we propose that the conceptual extension of models of complex cells with regard to an expansion over the degree of elongation of the receptive fields ought to generalize to also other types of functional models of complex cells.

More generally, we propose that the presented methodology made use of in this paper, of subjecting computational models of complex cells to similar probing tests as used for probing the orientation selectivity properties of biological complex cells, is important for understanding the theoretical properties of the computational mechanisms that are used for modelling biological complex cells. In this way, we will specifically demonstrate that complementing previous energy models of complex cells based on Gaussian derivatives with a spatial integration stage, as well as making use of spatial derivatives up to order 4 as opposed to previous use of spatial derivatives only up to order 2, has the potential of leading to better explanatory properties of orientation selectivity histograms of biological neurons, compared to the previous modelling approaches in Lindeberg [14,16].

Another important aspect of the presented work is that the proposed models for complex cells are based on theoretically well-founded models of simple cells, and specifically with very few free parameters to determine. By this *functional* modelling of the receptive fields at a coarse *macroscopic* level, the need for determining hyperparameters of the models is far lower compared what would be the case if one would instead base the analysis on more fine-grained models based on explicit neural models.

Thereby, the simulation work needed to reveal the qualitative properties of the computational models also becomes far lower compared to explicit simulations of networks of neural models, for which the result may also depend on the settings of the hyperparameters, and for which there may not be sufficient neurophysiological data available to tune the hyperparameters in a well-founded manner.

A general biological motivation for this study is that biological experiments often tend to reveal a variability of neurons in a variety of different respects. Connectivity analysis of the anatomy also tend to reveal a convergence along axonal projections. In this treatment, for idealized models of complex cells, we explain why there ought to be a variability in the degree of elongation, because of desirable affine covariance properties of an idealized vision system. Specifically, we demonstrate that this hypothesis is consistent with experimental results regarding orientation selectivity properties of biological neurons.

The theory of affine Gaussian derivative operators used as spatial models of the receptive fields also gives a theoretical motivation for the use of Gaussian derivative operators for different orders of spatial differentiation, with different numbers of main lobes in the receptive fields as function of the order of spatial differentiation. In combination with orientation selectivity histograms over the resultant of the orientation selectivity distributions for different orders of spatial differentiation, we demonstrate that the formulation of idealized models of complex cells based on a richer set of spatial derivatives leads to more uniform orientation selectivity histograms with closer similarity to orientation selectivity histograms accumulated from biological neurons.

In these ways, we demonstrate that the proposed computational mechanisms in terms of (i) a variability over the degree of elongation of the receptive fields, (ii) the combination of receptive field components corresponding to a richer set of orders of spatial differentiation, and (iii) the inclusion of explicit mechanisms for spatial integration provide ways towards bridging the gap between theoretical models of neural computation in relation to experimental results obtained from neurophysiological recordings of biological neurons.

Finally, we will use the results from the presented treatment (i) for stating a set of more general predictions in the section “Explicit predictions for further modelling of complex cells”, to be used as guide for further modelling of biological complex cells by mathematical models, and (ii) proposing a conceptual extension of the previous methodology for probing the orientation selectivity of biological neurons. The latter extension consists of instead of just recording the result for a single angular frequency for each image orientation instead performing a two-parameter variation over both the angular frequency and the image orientation, to be able to better reflect a variability in the degree of elongation between different individual biological neurons.

While the presented computational mechanisms are in the paper technically developed for the proposed family of affine quasi quadrature models of complex cells, we argue that corresponding additions of such computational mechanisms could be considered also for other types of theoretical and computational models of complex cells.

## Methods

### Related work

Orientation selectivity properties of biological neurons have been studied by Watkins and Berkley [17], Rose and Blakemore [18], Schiller et al. [19], Albright [20], Ringach et al. [21], Nauhaus et al. [22], Scholl et al. [23], Sadeh and Rotter [24], Goris et al. [15] and Sasaki et al. [25]. Biological mechanisms for achieving orientation selectivity have also been investigated by Somers et al. [26], Sompolinsky and Shapley [27], Carandini and Ringach [28], Lampl et al. [29], Ferster and Miller [30], Shapley et al. [31], Seriès et al. [32], Hansel and van Vreeswijk [33], Moldakarimov et al. [34], Gonzalo Cogno and Mato [35], Priebe [36], Pattadkal et al. [37], Nguyen and Freeman [38], Merkt et al. [39], Wei et al. [40] and Wang et al. [41]. In this paper, our focus is, however, not on neural mechanisms, but on *functional properties* at a macroscopic level.

Receptive field models of simple in terms of Gaussian derivatives have been formulated by Koenderink and van Doorn [42–44], Young and his co-workers [45–47] and Lindeberg [5,48], and in terms of Gabor functions by Marcelja [49], Jones and Palmer [50,51] and Porat and Zeevi [52]. More extensive theoretical models based on Gaussian derivatives have also been expressed by Lowe [53], May and Georgeson [54], Hesse and Georgeson [55], Georgeson et al. [56], Hansen and Neumann [57], Wallis and Georgeson [58], Wang and Spratling [59], Pei et al. [60], Ghodrati et al. [61], Kristensen and Sandberg [62], Abballe and Asari [63], Ruslim et al. [64] and Wendt and Faul [65].

The taxonomy into simple and complex cells in the primary visual cortex was proposed in the pioneering work by Hubel and Wiesel [1–4]. More extensive analysis of properties of simple cells have then been presented by DeAngelis et al. [6,7], Ringach [66,67], Conway and Livingstone [8], Johnson et al. [9], Walker et al. [68] and De and Horwitz [69], and regarding complex cells by Movshon et al. [70], Emerson et al. [71], Martinez and Alonso [72], Touryan et al. [73,74], Rust et al. [75], van Kleef et al. [76], Goris et al. [15], Li et al. [77] and Almasi et al. [78], as well as modelled computationally by Adelson and Bergen [79], Heeger [80], Serre and Riesenhuber [81], Einhäuser et al. [82], Kording et al. [83], Merolla and Boahen [84], Berkes and Wiscott [85], Carandini [86], Hansard and Horaud [87], Franciosini et al. [88], Lindeberg [89], Lian [90], Oleskiw et al. [91], Yedjour and Yedjour [92], Nguyen et al. [93] and Almasi et al. [94].

Notably, in relation to the generalized quadratic models of complex cells in V1 to be considered in this paper, Rowekamp and Sharpee [95] have found that quadratic computations strongly increase both the predictive power of their models of visual neurons in V1, V2 and V4 as well as their neural selectivity to natural stimuli.

There have been some neurophysiological studies reported that mention receptive fields with different aspect ratios (Tinsley et al. [96], Xu et al. [97]). According to our knowledge, there have, however, not been any previously developed models of complex cells, that involve explicit expansions of the receptive field shapes over the degree of elongation of the receptive fields, or or more specifically that are able to match the neurophysiological orientation selectivity histograms accumulated by Goris et al. [15].

### Background theory

In this section, we will describe basic properties of the idealized models for idealized models of receptive fields and their orientation selectivity properties, which we will then build upon and extend in the section “Results”.

#### Idealized models for spatial receptive fields.

For modelling simple and complex cells in the primary visual cortex, we will build upon the generalized Gaussian derivative model for visual receptive fields proposed in Lindeberg [5,48] and further developed in Lindeberg [10,12,14,16].

#### Models for simple cells.

According to this theory, linear models of purely spatial receptive fields corresponding to simple cells are formulated in terms of affine Gaussian derivatives of the form

$$T_{\text{simple}}(x_{1},x_{2};\;\sigma_{\varphi },\varphi ,\Sigma_{\varphi },m)=\;=T_{\varphi^{m},\text{norm}}(x_{1},x_{2};\;\sigma_{\varphi },\Sigma_{\varphi })=\sigma_{\varphi }^{m}\;\partial_{\varphi }^{m}\left(g(x_{1},x_{2};\;\Sigma_{\varphi })\right),$$ (1)

where

- φ∈[−π,π] is the preferred orientation of the receptive field,
- $\sigma_{\varphi }\in R_{+}$ is the amount of spatial smoothing,
- $\partial_{\varphi }^{m}=(\cos\varphi \;\partial_{x_{1}}\;+\;\sin\varphi \;\partial_{x_{2}}{)}^{m}$ is an *m*:th-order directional derivative operator, in the direction φ,
- $\Sigma_{\varphi }$ is a 2×2 symmetric positive definite covariance matrix, with one of its eigenvectors in the direction of φ,
- $g(x;\;\Sigma_{\varphi })$ is a 2-D affine Gaussian kernel with its shape determined by the spatial covariance matrix $\Sigma_{\varphi }$
  $$g(x;\;\Sigma_{\varphi })=\frac{1}{2\pi \sqrt{\det\Sigma_{\varphi }}}e^{-x^{T}\Sigma_{\varphi }^{-1}x/2}$$ (2)
  for $x=(x_{1},x_{2}{)}^{T}\in R^{2}$.

In Lindeberg [5], it was demonstrated that idealized receptive field models of this type do rather well model the qualitative shape of biological simple cells as obtained by neurophysiological measurements by DeAngelis et al. [6,7], Conway and Livingstone [8] and Johnson et al. [9].

**Fig 1:**
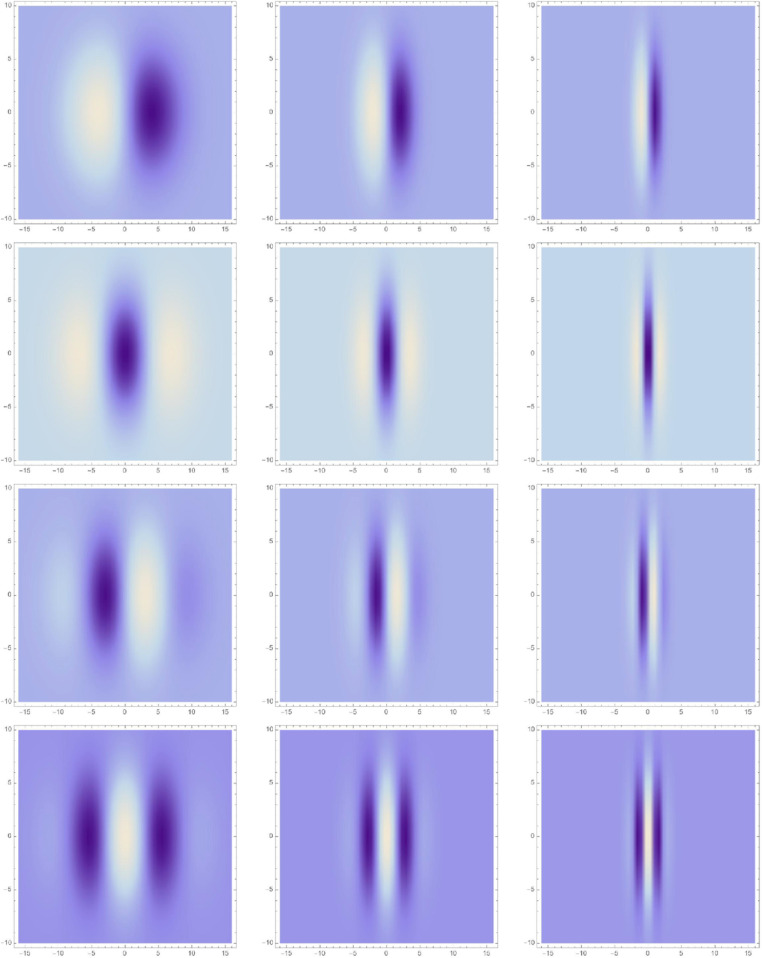
Distribution of affine Gaussian derivative receptive fields (for the preferred image orientation φ=0) over the scale parameter ratio $\kappa =\sigma_{2}/\sigma_{1}$ between 1 to 4, from left to right. Here, the vertical scale parameter kept is constant $\sigma_{2}=4$, while the horizontal scale parameter is smaller $\sigma_{1}\leq \sigma_{2}$. (first row) First-order directional derivatives according to (1) for *m* = 1. (second row) Second-order directional derivatives according to (1) for *m* = 2. (third row) Third-order directional derivatives according to (1) for *m* = 3. (fourth row) Fourth-order directional derivatives according to (1) for *m* = 4. (Horizontal axes: image coordinate $x_{1}\in [-16,16]$. Vertical axes: image coordinate $x_{2}\in [-16,16]$.)

Fig 1 shows examples of such receptive fields for different orders of spatial differentiation m∈{1,2,3,4} and different values of the scale parameter ratio $\kappa =\sigma_{2}/\sigma_{1}\in \{1,2,4\}$ between the here vertical and the horizontal scale parameters $\sigma_{2}$ and $\sigma_{1}$, respectively, for the here preferred orientation φ=0 for the receptive fields. In addition to this illustrated variability over the degree of elongation, an idealized vision system should additionally comprise a variability over the preferred orientation φ of the receptive fields, and possibly also over the overall size of the receptive fields, as further developed in Lindeberg [14].

#### Models for complex cells.

As a simplest possible extension to non-linear complex cells, an affine quasi-quadrature measure of the form (Lindeberg [89] Eq (39))

$${𝒬}_{\varphi ,12,\text{pt}}L=\sqrt{L_{\varphi ,\text{norm}}^{2}+C_{\varphi }\;L_{\varphi \varphi ,\text{norm}}^{2}},$$ (3)

was studied in Lindeberg [14], where

- $L_{\varphi ,\text{norm}}$ and $L_{\varphi \varphi ,\text{norm}}$ denote directional derivatives in the direction φ of orders 1 and 2 of convolutions of the input image $f(x_{1},x_{2})$ with affine Gaussian derivative kernels of the form (1):
  $$L_{\varphi ,\text{norm}}(x_{1},x_{2};\;\sigma_{\varphi },\Sigma_{\varphi })=T_{\varphi ,\text{norm}}(x_{1},x_{2};\;\sigma_{\varphi },\Sigma_{\varphi })*f(x_{1},x_{2}),$$ (4)
  $$L_{\varphi \varphi ,\text{norm}}(x_{1},x_{2};\;\sigma_{\varphi },\Sigma_{\varphi })=T_{\varphi \varphi ,\text{norm}}(x_{1},x_{2};\;\sigma_{\varphi },\Sigma_{\varphi })*f(x_{1},x_{2}),$$ (5)
- $C_{\varphi }>0$ is a weighting factor between first and second-order information, which based on a theoretical analysis in Lindeberg [98] is often set to $C=1/\sqrt{2}$.

This model is closely related to the energy model of complex cells proposed by Adelson and Bergen [79] and Heeger [80], as well as inspired by the fact that odd- and even-shaped receptive fields have been reported to occur in pairs (De Valois et al. [99]). The quasi quadrature serves as an approximation of a quadrature pair, as formulated based on a Hilbert transform (Bracewell [100], pp. 267–272), although instead formulated in terms of affine Gaussian derivatives, which are then summed up in squares in to reduce the phase dependency; see Lindeberg [89] for further details.

Concerning the validity of such an energy-based model of simple cells to model the computational function of complex cells, it is interesting to note that when Touryan et al. [74] extracted the eigenvectors of second-order Wiener kernels to model the computational function of complex cells, the first two eigenvectors turned out the have spatial shapes that very well agree with the shapes of first- and second-order Gaussian derivatives; compare with Fig 5B in Touryan et al. [74]. Hence, even though an energy model of complex cells in terms of the output from a set of simple cells may not be able to span the full flexibility in terms of the possible computational functions of biological complex cells, an approximation of the computational function of a complex cell in terms of an energy model in such a way ought to be able to reveal essential properties of the computational functionalities of complex cells.

#### Orientation selectivity properties.

**Fig 2:**
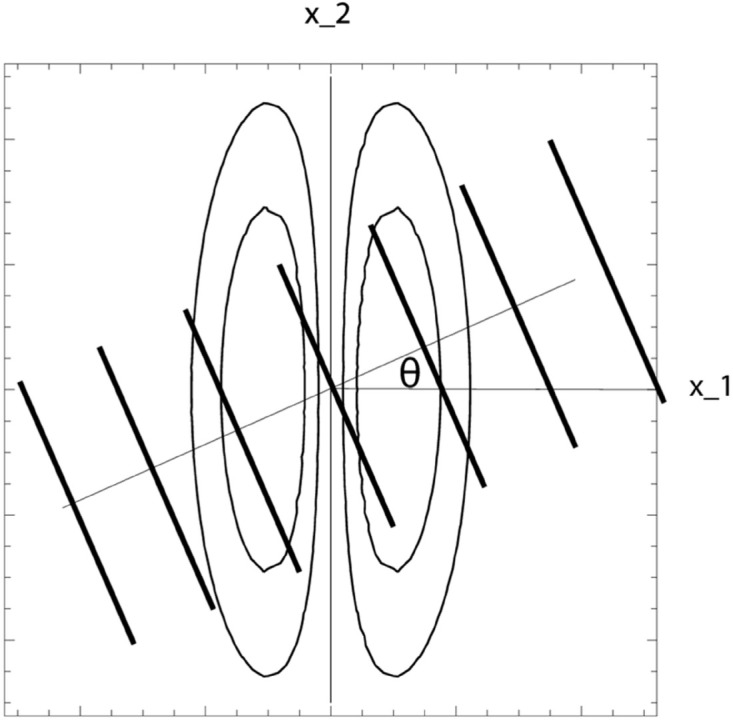
Configuration for probing the orientation selectivity of an idealized receptive field model. To measure the orientation selectivity properties of an idealized receptive field (here illustrated by the level curves of a first-order derivative of an affine Gaussian kernel in the horizontal direction with preferred orientation φ=0), we compute the response to a sine wave (here illustrated by a set of darker level lines corresponding to the spatial maxima and minima of the sine wave) with inclination angle *θ*. (Horizontal axis: spatial coordinate *x*_1_. Vertical axis: spatial coordinate *x*_2_.) (Adapted from Lindeberg [16] OpenAccess.)

In Lindeberg [14,16], the orientation selectivity properties of these idealized models of simple and complex cells were investigated in detail, by computing the responses to sine wave functions of the form

$$f(x_{1},x_{2})=\sin\left(\omega \cos(\theta )\;x_{1}+\omega \sin(\theta )\;x_{2}+\beta \right)$$ (6)

where θ∈[−π/2,π/2] denotes the inclination angle of the sine wave in relation to the preferred orientation φ=0 of the receptive field and *β* denotes the phase; see Fig 2 for an illustration.

Specifically, in Lindeberg [14,16], it was shown that with

$$\kappa =\frac{\sigma_{2}}{\sigma_{1}}$$ (7)

denoting the ratio between the scale parameters $\sigma_{1}\in R_{+}$ and $\sigma_{2}\in R_{+}$ of the affine Gaussian kernel in the preferred direction *vs.* the orthogonal direction of the receptive field, the orientation selectivity curves are of the form

$$r_{\lambda }(\theta )={\left(\frac{\left|\cos\theta \right|}{\sqrt{\cos^{2}\theta +\kappa^{2}\sin^{2}\theta }}\right)}^{\lambda }$$ (8)

with

- λ=m for an *m*:th-order model of a simple cell of the form (1) for m∈{1,2,3,4} and
- λ=3/2 for an idealized model of a complex cell of the form (3).

Notably, for all these idealized models of the receptive fields, a smaller value of the scale parameter ratio κ leads to wider orientation selectivity curves, whereas larger values of κ lead to more narrow orientation selectivity properties. In this respect, given the assumption that the affine Gaussian derivative model should constitute an appropriate model for the spatial component of simple cells, a variability in the degree of elongation of the receptive fields will correspond to a variability in the orientation selectivity and *vice versa*.

## Results

### Generalized idealized models of complex cells

In this work, we will extend the idealized model (3) for complex cells in two major ways:

- by adding a spatial integration stage, and
- considering derivatives up to order 4 in addition to derivatives up to order 2.

The motivation for adding a spatial integration stage is that the model for complex cell should then make use of input from more than two simple cells, specifically by accumulating input over multiple positions in the visual field.

The motivation for adding derivatives of higher order than 2 are: (i) to enable more narrow orientation selectivity properties and (ii) in Lindeberg [14] it was shown that models of simple cells up to order 4 lead to better agreement with the orientation selectivity histogram of simple cells recorded by Goris et al. [15] than simple cells up to order 2.

Thus, if we let $L_{\varphi \varphi \varphi ,\text{norm}}$ and $L_{\varphi \varphi \varphi \varphi ,\text{norm}}$ denote the directional derivatives in the direction φ of orders 3 and 4 of convolutions of the input image $f(x_{1},x_{2})$ with affine Gaussian derivative kernels of the form (1):

$$L_{\varphi \varphi \varphi ,\text{norm}}(x_{1},x_{2};\;\sigma_{\varphi },\Sigma_{\varphi })=T_{\varphi \varphi \varphi ,\text{norm}}(x_{1},x_{2};\;\sigma_{\varphi },\Sigma_{\varphi })*f(x_{1},x_{2}),$$ (9)

$$L_{\varphi \varphi \varphi \varphi ,\text{norm}}(x_{1},x_{2};\;\sigma_{\varphi },\Sigma_{\varphi })=T_{\varphi \varphi \varphi \varphi ,\text{norm}}(x_{1},x_{2};\;\sigma_{\varphi },\Sigma_{\varphi })*f(x_{1},x_{2}),$$ (10)

as well as consider the previous definitions of $L_{\varphi ,\text{norm}}$ and $L_{\varphi \varphi ,\text{norm}}$ according to (4) and (5), we will consider the following new generalized and integrated affine quasi quadrature measures for modelling complex cells:

$${𝒬}_{\varphi ,12,\text{int}}L=\sqrt{\sum_{m\in \{1,2\}}g(\cdot ,\cdot ;\;\gamma^{2}\;\Sigma_{\varphi })*C_{\varphi }^{m-1}\;L_{\varphi^{m},\text{norm}}^{2}(\cdot ,\cdot ;\;\sigma_{\varphi },\Sigma_{\varphi })}$$ (11)

$${𝒬}_{\varphi ,1234,\text{int}}L=\sqrt{\sum_{m\in \{1,2,3,4\}}g(\cdot ,\cdot ;\;\gamma^{2}\;\Sigma_{\varphi })*C_{\varphi }^{m-1}\;L_{\varphi^{m},\text{norm}}^{2}(\cdot ,\cdot ;\;\sigma_{\varphi },\Sigma_{\varphi })}$$ (12)

$${𝒬}_{\varphi ,34,\text{int}}L=\sqrt{\sum_{m\in \{3,4\}}g(\cdot ,\cdot ;\;\gamma^{2}\;\Sigma_{\varphi })*C_{\varphi }^{m-3}\;L_{\varphi^{m},\text{norm}}^{2}(\cdot ,\cdot ;\;\sigma_{\varphi },\Sigma_{\varphi })},$$ (13)

where $g(\cdot ,\cdot ;\;\gamma^{2}\;\Sigma_{\varphi })$ for the relative integration scale γ>1 denotes a spatially larger affine Gaussian kernel than the affine Gaussian kernel $g(\cdot ,\cdot ;\;\Sigma_{\varphi })$ used for computing the receptive field responses $L_{\varphi^{m},\text{norm}}$ for the idealized models of simple cells. In the following experiments to be reported, we will throughout use the parameter setting $\gamma =1/\sqrt{2}$.

Structurally, these expressions are similar in the sense that the squares of the directional derivative responses $L_{\varphi^{m},\text{norm}}$ are first integrated for different orders *m* of spatial differentiation, and then summed of for different subsets m∈{1,2}, m∈{1,2,3,4} and m∈{3,4}, respectively. Specifically, the first of these integrated affine quasi quadrature measures ${𝒬}_{\varphi ,12,\text{int}}L$ can basically be seen as a spatially integrated extension of the pointwise affine quasi quadrature measure ${𝒬}_{\varphi ,12,\text{pt}}L$ in (3).

### Orientation selectivity curves for the generalized idealized models of complex cells

To characterize the orientation selectivity properties for the generalized integrated affine quasi quadrature measures (11), (12) and (13), let us first compute the responses $L_{\varphi^{m},\text{norm}}$ for the underlying models of simple cells $T_{\varphi^{m},\text{norm}}$ to a sine wave (6) according to Eqs (29) and (35) in Lindeberg [16]

$$L_{0,\text{norm}}(x_{1},x_{2};\;\sigma_{1},\sigma_{2})=\;=\int_{\xi_{1}=-\infty }^{\infty }\int_{\xi_{2}=-\infty }^{\infty }T_{0,\text{norm}}(\xi_{1},\xi_{2};\;\sigma_{1},\sigma_{2})\;f(x_{1}-\xi_{1},x_{2}-\xi_{2})\;d\xi_{1}\;d\xi_{2}\;=\omega \;\sigma_{1}\cos(\theta )\;e^{-\frac{1}{2}\omega^{2}(\sigma_{1}^{2}\cos^{2}\theta +\sigma_{2}^{2}\sin^{2}\theta )}\;\cos(\omega \cos(\theta )\;x_{1}+\omega \sin(\theta )\;x_{2}+\beta ),$$ (14)

$$L_{00,\text{norm}}(x_{1},x_{2};\;\sigma_{1},\sigma_{2})=\;=\int_{\xi_{1}=-\infty }^{\infty }\int_{\xi_{2}=-\infty }^{\infty }T_{00,\text{norm}}(\xi_{1},\xi_{2};\;\sigma_{1},\sigma_{2})\;f(x_{1}-\xi_{1},x_{2}-\xi_{2})\;d\xi_{1}\;d\xi_{2}\;=-\omega^{2}\;\sigma_{1}^{2}\cos^{2}(\theta )\;e^{-\frac{1}{2}\omega^{2}(\sigma_{1}^{2}\cos^{2}\theta +\sigma_{2}^{2}\sin^{2}\theta )}\;\sin(\omega \cos(\theta )\;x_{1}+\omega \sin(\theta )\;x_{2}+\beta ),$$ (15)

and according to Eqs (36) and (42) in the supplementary material of Lindeberg [14]

$$L_{000,\text{norm}}(x_{1},x_{2};\;\sigma_{1},\sigma_{2})=\;=\int_{\xi_{1}=-\infty }^{\infty }\int_{\xi_{2}=-\infty }^{\infty }T_{000,\text{norm}}(\xi_{1},\xi_{2};\;\sigma_{1},\sigma_{2})\;f(x_{1}-\xi_{1},x_{2}-\xi_{2})\;d\xi_{1}\;d\xi_{2}\;=-\omega^{3}\;\sigma_{1}^{3}\cos^{3}(\theta )\;e^{-\frac{1}{2}\omega^{2}(\sigma_{1}^{2}\cos^{2}\theta +\sigma_{2}^{2}\sin^{2}\theta )}\;\cos(\omega \cos(\theta )\;x_{1}+\omega \sin(\theta )\;x_{2}+\beta ),$$ (16)

$$L_{0000,\text{norm}}(x_{1},x_{2};\;\sigma_{1},\sigma_{2})=\;=\int_{\xi_{1}=-\infty }^{\infty }\int_{\xi_{2}=-\infty }^{\infty }T_{0000,\text{norm}}(\xi_{1},\xi_{2};\;\sigma_{1},\sigma_{2})\;f(x_{1}-\xi_{1},x_{2}-\xi_{2})\;d\xi_{1}\;d\xi_{2}\;=\omega^{4}\;\sigma_{1}^{4}\cos^{4}(\theta )\;e^{-\frac{1}{2}\omega^{2}(\sigma_{1}^{2}\cos^{2}\theta +\sigma_{2}^{2}\sin^{2}\theta )}\;\sin(\omega \cos(\theta )\;x_{1}+\omega \sin(\theta )\;x_{2}+\beta ),$$ (17)

where $\sigma_{1}\in R_{+}$ and $\sigma_{2}\in R_{+}$ denote the scale parameters of the affine Gaussian kernel in the horizontal and the vertical directions, respectively.

Let us next integrate the squares of these expressions spatially using a Gaussian window function with relative integration scale γ>1, with the actual calculations performed in Wolfram Mathematica and leading to results that are unfortunately too complex to be reproduced here.

(A Wolfram Mathematica notebook with the commands for computing these integrals in closed form, and more generally also generating all the results figures in this paper, including (i) the orientation selectivity curves r(φ), (ii) the resultant measures |R(κ)| for different degrees of elongation κ, and (iii) the orientation selectivity histograms of the resultant |R| for the different models for complex cells considered in this paper, is provided as supplementary information “S2 File”.)

Let us furthermore for the parameterization of the vertical scale parameter $\sigma_{2}=\kappa \;\sigma_{1}$ consider the angular frequencies $\hat{\omega_{1}}$, $\hat{\omega_{2}}$, $\hat{\omega_{3}}$ and $\hat{\omega_{4}}$ for which these expressions assume their maxima over angular frequencies according to Eqs (33) and (38) in Lindeberg [16]

$${\hat{\omega }}_{\varphi }=\frac{1}{\sigma_{1}\sqrt{\cos^{2}\theta +\kappa^{2}\sin^{2}\theta }},$$ (18)

$${\hat{\omega }}_{\varphi \varphi }=\frac{\sqrt{2}}{\sigma_{1}\sqrt{\cos^{2}\theta +\kappa^{2}\sin^{2}\theta }},$$ (19)

and according to Eqs (40) and (45) in the supplementary material of Lindeberg [14]

$${\hat{\omega }}_{\varphi \varphi \varphi }=\frac{\sqrt{3}}{\sigma_{1}\sqrt{\cos^{2}\theta +\kappa^{2}\sin^{2}\theta }},$$ (20)

$${\hat{\omega }}_{\varphi \varphi \varphi \varphi }=\frac{2}{\sigma_{1}\sqrt{\cos^{2}\theta +\kappa^{2}\sin^{2}\theta }}.$$ (21)

Given these preferred angular frequencies for the different orders of spatial differentiation, let us next for the composed integrated affine quasi quadrature measures (11), (12) and (13) choose the geometric averages of the respective components according to

$${\hat{\omega }}_{12}=\sqrt{{\hat{\omega }}_{\varphi }\;{\hat{\omega }}_{\varphi \varphi }},$$ (22)

$${\hat{\omega }}_{1234}=\sqrt{{\hat{\omega }}_{\varphi }\;{\hat{\omega }}_{\varphi \varphi }\;{\hat{\omega }}_{\varphi \varphi \varphi }\;{\hat{\omega }}_{\varphi \varphi \varphi \varphi }},$$ (23)

$${\hat{\omega }}_{34}=\sqrt{{\hat{\omega }}_{\varphi \varphi \varphi }\;{\hat{\omega }}_{\varphi \varphi \varphi \varphi }},$$ (24)

and adapt the angular frequency of the probing sine wave in these ways to the respective quasi quadrature measures ${𝒬}_{\varphi ,12,\text{int}}L$ according to (11), ${𝒬}_{\varphi ,1234,\text{int}}L$ according to (12) and ${𝒬}_{\varphi ,34,\text{int}}L$ according to (13). This adaptation of the angular frequency of the sine wave to the internal parameters of the model of the complex cell corresponds to probing the corresponding complex cells for different values of the angular frequency *ω* and then choosing the orientation selectivity curve for the angular frequency $\hat{\omega }$ that leads to the maximum response over all the frequencies *ω*.

**Fig 3:**
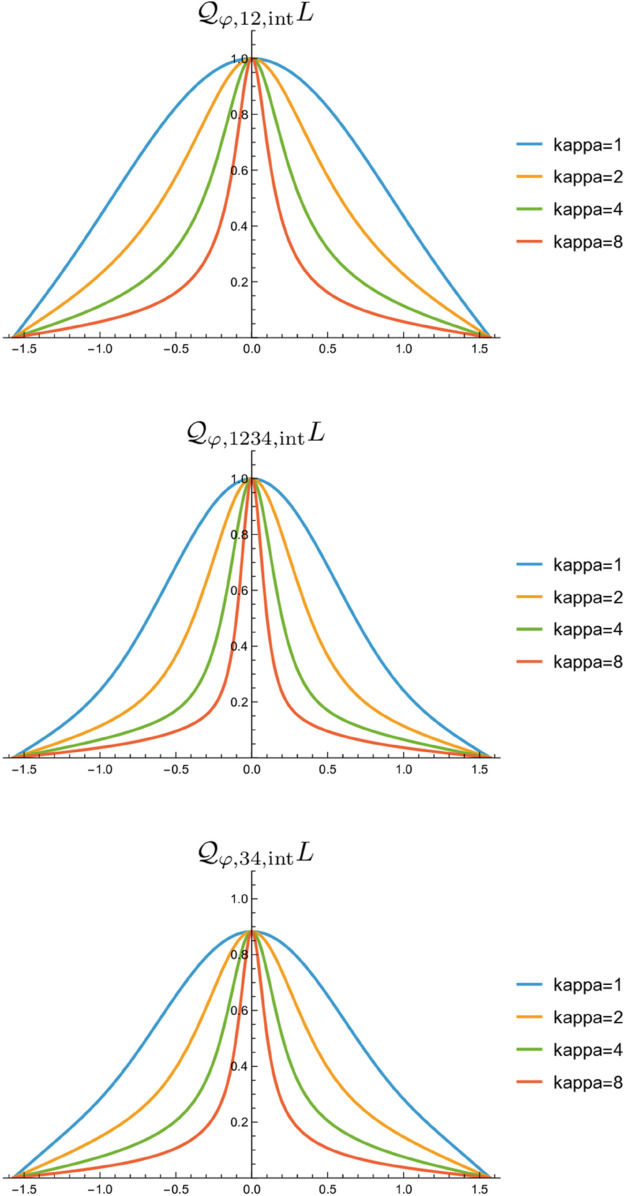
Graphs of the orientation selectivity curves for the generalized integrated affine quasi quadrature measures ${𝒬}_{\varphi ,12,\text{int}}L$ according to (11), ${𝒬}_{\varphi ,1234,\text{int}}L$ according to (12) and ${𝒬}_{\varphi ,34,\text{int}}L$ according to (13), and which combine integrated squared values of affine Gaussian derivative responses for different combinations of orders of integration m∈{1,2,3,4} when applied to an ideal sine wave of the form (6) with the angular frequency of the sine wave adapted so as to evoke a maximally strong response over the angular frequencies according to (22), (23) and (24), respectively. The resulting orientation selectivity curves are shown for different values of the scale parameter ratio $\kappa =\sigma_{2}/\sigma_{1}\in \{1,2,4,8\}$, which parameterizes the degree of elongation of the receptive fields. (Horizontal axes: orientation θ∈[−π/2,π/2]. Vertical axes: Amplitude of the receptive field response relative to the maximum response obtained for θ=0.)

Fig 3 shows the resulting orientation selectivity curves that we then obtain for different values of the scale parameter ratio κ, when using the relative integration scale $\gamma =1/\sqrt{2}$ for the spatial integration stage and the weighting factor $C=1/\sqrt{2}$ when combining the responses for different orders *m* of differentiation. As can be seen from these graphs, for all the three generalized integrated affine quasi quadrature measures, the orientation selectivity curves become sharper with increasing values of the scale parameter ratio κ. In this respect, these results are consistent with the previously reported results in Lindeberg [14,16].

Specifically, in relation to the previously recorded orientation selectivity curves for biological neurons reported in Nauhaus et al. [22], and which span a variability in orientation selectivity from wide to narrow orientation selectivity properties, these results are consistent with what would be the case if the receptive fields in the primary visual cortex would span a variability in the degree of elongation, as proposed as a working hypothesis in Lindeberg [10] Sect 3.2.1 and further investigated in Lindeberg [14].

### Orientation selectivity histograms for the generalized idealized models of complex cells

The previous analysis is *qualitative* in the sense that it shows that the orientation selectivity curves become sharper for increasing values of the scale parameter ratio κ, and also in the respect that a variability in the orientation selectivity properties is consistent with an underlying variability in the degree of elongation of the receptive fields.

To aim at a more *quantitative* analys, let us compare the result of our idealized integrated models of complex cells with the quantitative measurements of orientation selectivity histograms reported by Goris et al. [15]. They accumulated histograms of the absolute value of the resultant |R| with the underlying complex-valued resultant of an orientation selectivity curve r(θ) of the form

$$R=\frac{\int_{\theta =-\pi }^{\pi }r(\theta )\;e^{2i\theta }d\theta }{\int_{\theta =-\pi }^{\pi }r(\theta )\;d\theta }.$$ (25)

**Fig 4:**
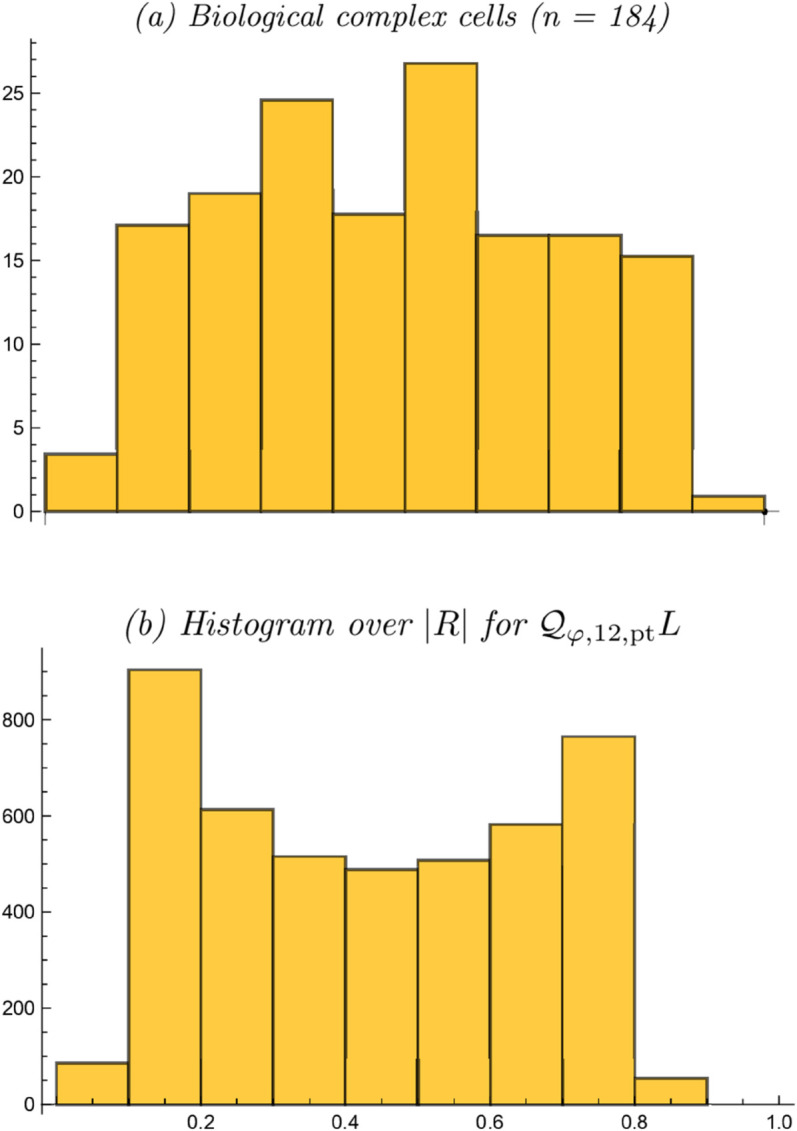
Orientation selectivity histogram of the resultant of biological complex cells accumulated by Goris et al. [15] with comparison to histogram of the resultant from previous orientation selectivity analysis of maximally simplified models of complex cells in Lindeberg [14]. (Horizontal axes: 10 quantized bins over the resultant |R|∈[0,1]. Vertical axes: bin counts.)

Fig 4a gives a schematic illustration of the results that they obtained, reflecting a significant variability in wide *vs.* narrow orientation selectivity properties for different biological complex cells. Fig 4b shows a corresponding prediction of a histogram of the resultant of the orientation selectivity curves obtained in Lindeberg [14], based on the pointwise quasi quadrature measure ${𝒬}_{\varphi ,12,\text{pt}}L$ in (3), and assuming that the scale parameter ratio κ would be uniformly distributed on a logarithmic scale over the interval $\left[1/\kappa_{\max},\kappa_{max}\right]$ for the arbitrary choice of $\kappa_{max}=8$.

As can be seen from the comparison between the biological results and the idealized modelling results, the distribution over the resultant |R| is somewhat biased towards both smaller and larger values of |R|, specifically regarding the peaks in the histogram at the bins |R|∈[0.1,0.2] and |R|∈[0.7,0.8], compared to the neurophysiologically accumulated resultant histograms. A natural question to ask is hence if this behaviour would be different if using more developed models of complex cells, that also comprise a spatial integration stage and derivatives of higher order than 2.

**Fig 5:**
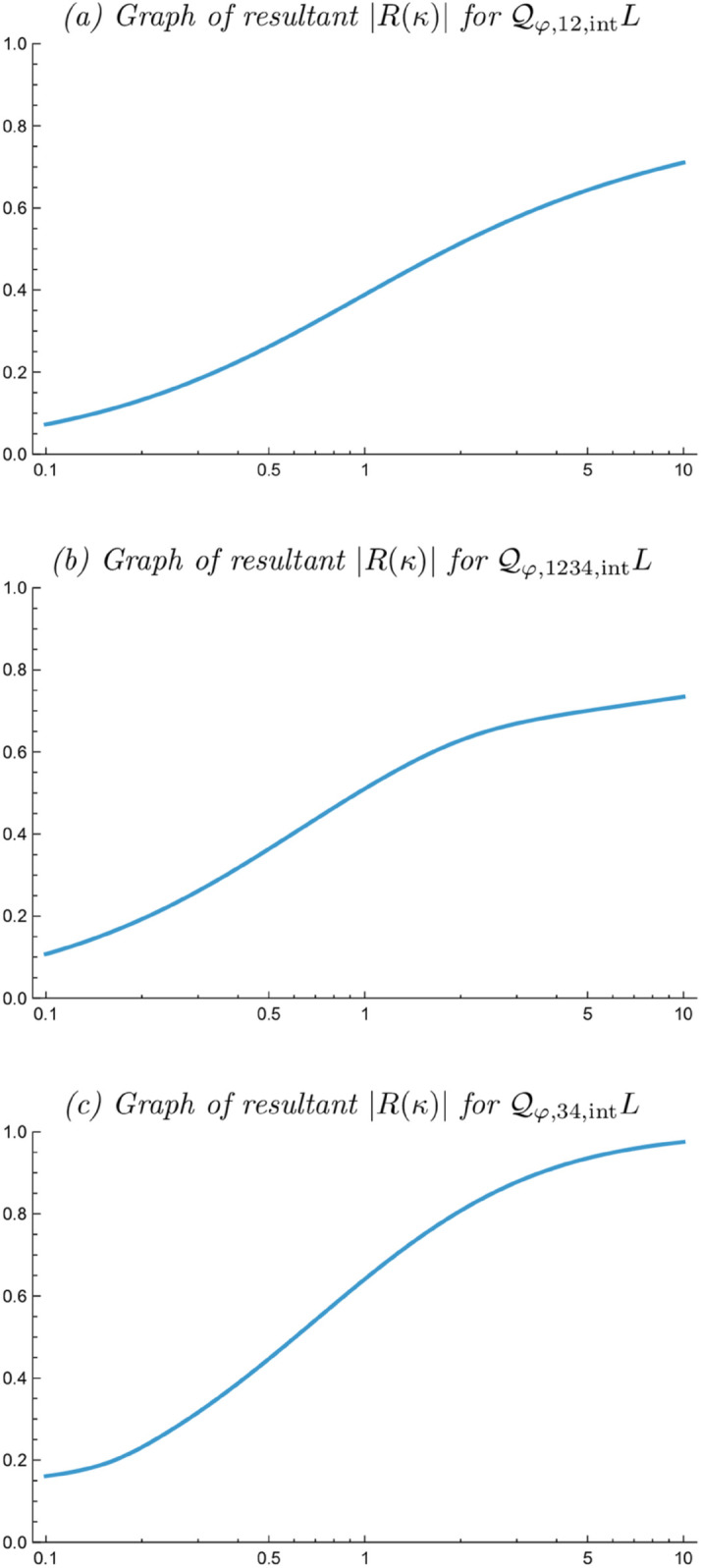
Graphs of the resultant |R(κ)| for the idealized models of complex cells ${𝒬}_{\varphi ,12,\text{int}}L$ according to (11), ${𝒬}_{\varphi ,1234,\text{int}}L$ according to (12) and ${𝒬}_{\varphi ,34,\text{int}}L$ according to (13). (Horizontal axes in: scale parameter ratio κ. Vertical axes: resultant |R|.)

Fig 5 shows the result of computing the resultant measure |R| as a function of the scale parameter ratio $\kappa =\sigma_{2}/\sigma_{1}$ for each one of the integrated affine quasi quadrature measures ${𝒬}_{\varphi ,12,\text{int}}L$, ${𝒬}_{\varphi ,1234,\text{int}}L$ and ${𝒬}_{\varphi ,34,\text{int}}L$ according to (11), (12) and (13).

**Fig 6:**
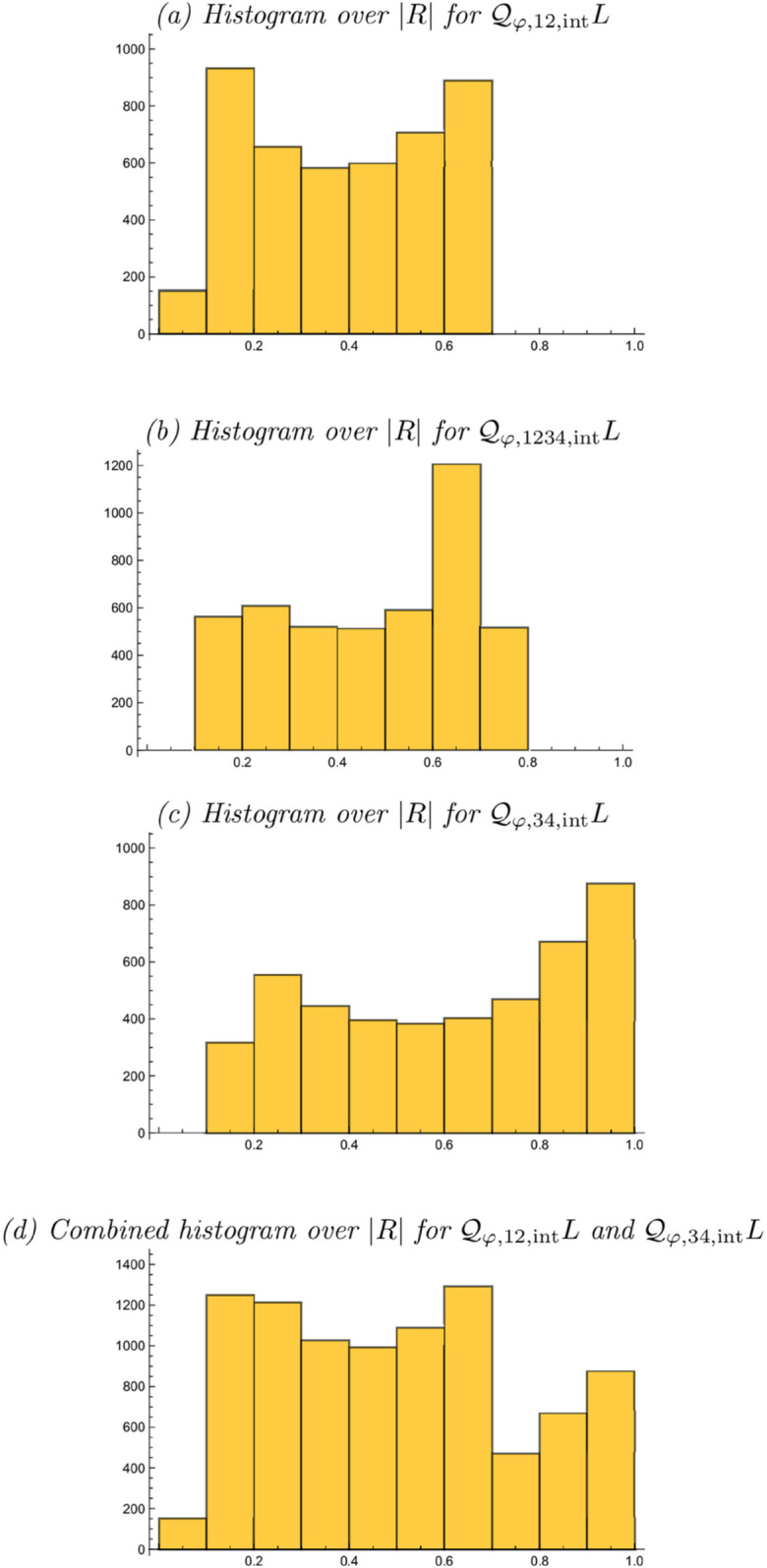
Orientation selectivity histograms for the resultant |R| for (a–c) the integrated affine quasi quadrature measures ${𝒬}_{\varphi ,12,\text{int}}L$, ${𝒬}_{\varphi ,1234,\text{int}}L$ and ${𝒬}_{\varphi ,34,\text{int}}L$ according to (11), (12) and (13), when assuming a uniform distribution over a logarithmic parameterization of the scale parameter ratio κ, as well as (d) combined histogram of ${𝒬}_{\varphi ,12,\text{int}}L$ and ${𝒬}_{\varphi ,34,\text{int}}L$, when assuming equal numbers of complex cells for these two types. (Horizontal axes: 10 quantized bins over the resultant |R|∈[0,1]. Vertical axes: bin counts.)

Fig 6a–6c show corresponding resultant histograms for each of these integrated affine quasi quadrature measures, when assuming a uniform distribution over a logarithmic parameterization of the scale parameter ratio κ over the interval $\left[1/\kappa_{\max},\kappa_{\max}\right]$ for the again arbitrary value of $\kappa_{\max}=8$. Such a logarithmic distribution constitutes a natural default prior for a strictly positive variable according to Jaynes [101]. Fig 6d shows a combined histogram of the integrated affine quasi quadrature measures ${𝒬}_{\varphi ,12,\text{int}}L$ and ${𝒬}_{\varphi ,34,\text{int}}L$, when assuming an equal number of idealized complex cells for these two types.

As can be seen from these histograms, the use of the integrated affine quasi quadrature measures ${𝒬}_{\varphi ,12,\text{int}}L$, ${𝒬}_{\varphi ,1234,\text{int}}L$ and ${𝒬}_{\varphi ,34,\text{int}}L$ leads to resultant histograms with different shapes than for pointwise quasi quadrature measure ${𝒬}_{\varphi ,12,\text{pt}}L$ previously studied in Lindeberg [14]. Specifically, except for the peak at the bin corresponding to |R|∈[0.6,0.7], the resultant histogram of ${𝒬}_{\varphi ,1234,\text{int}}L$ is more uniform and without any bias the bias towards either smaller values of the resultant |R| towards the bins |R|∈[0.1,0.2] and |R|∈[0.7,0.8] as for the resultant histogram of the pointwise quasi quadrature measure ${𝒬}_{\varphi ,12,\text{pt}}L$.

Additionally, when including third- and fourth-order terms, the integrated affine quasi quadrature measures ${𝒬}_{\varphi ,1234,\text{int}}L$ and ${𝒬}_{\varphi ,34,\text{int}}L$ lead to accumulations to the bins |R|∈[0.8,0.9] and |R|∈[0.9,1.0], while those bins are not really reached by the pointwise quasi quadrature measure ${𝒬}_{\varphi ,12,\text{pt}}L$. In these respects, this analysis suggests that the mechanisms of spatial integration and inclusion of higher-order terms than mere first- and second-order terms may be important to more quantitatively model the orientation selectivity properties of complex cells. Furthermore, one may speculate if a suitable reformulation of the non-linearities in the composed integrated affine quasi quadrature measure ${𝒬}_{\varphi ,1234,\text{int}}L$ could move the peak for the bin |R|∈[0.6,0.7] to the bin positions |R|∈[0.3,0.4] and |R|∈[0.5,0.6] that lead to peaks in the resultant histogram for the biological complex cells.

Based on these results, we therefore propose to (i) include an explicit variability over the degree of elongation of the receptive fields, (ii) include third- and fourth-order models of simple cells in addition to previous use of first- and second-order models of simple cells and (iii) integrate non-linear transformations of such receptive field responses over extended regions in image space, when modelling the functional properties of complex cells. Specifically, we propose that the items (i) and (iii) above should be of much wider generality than restricted to computational models based on affine quasi quadrature measures, and thereby also apply to other families of computational models of complex cells.

### Explicit predictions for further modelling of complex cells

More explicitly, based on the above results, we can thus state the following general predictions:

**Prediction 1 (Flexibility in elongation would lead to better approximation properties when modelling individual complex cells):** Given a sufficiently large set of biological complex cells, if the receptive fields of those are modelled by computational models, then such models that involve a flexibility in the degree of elongation of the underlying computational primitives would lead to better approximation properties than for computational models that do not involve a flexibility in the degree of elongation.

**Prediction 2 (Variability in elongation over populations of complex cells):** If the receptive fields sufficiently large set of complex cells are modelled by computational models that involve a flexibility in the degree of elongation of the underlying computational primitives, then such model fitting would lead to a substantial variability in the degree of elongation of the receptive fields over a sufficiently large population of modelled biological complex cells.

**Prediction 3 (Spatial integration as a computational mechanism in complex cells):** Given a sufficiently large set of biological complex cells, if their receptive fields are modelled by computational models, then models that are based on spatial integration of underlying computational primitives would lead to better approximation properties than computational models that do not comprise any spatial integration.

Specifically, we propose that these predictions could be explored and influence further modelling of complex cells in terms of mathematically based image primitives.

### Explicit suggestion to extension of methodology for experimentally probing the orientation selectivity of complex cells that may comprise a variability in the degree of elongation between different visual neurons.

Furthermore, to allow for better distinctions between the validity of different types of computational models for complex cells, we propose to extend the experimental methodology for measuring the orientation selectivity properties of biological neurons to instead of (i) choosing a single angular frequency for the probing sine wave for generating the visual stimuli for each spatial image orientation, instead (ii) performing a simultaneous *two-parameter variation* over both the angular frequency and the image orientation. In such a way, the experimental data ought to be better suited for handling biological receptive fields with a variability in elongation. The reason for this, is that the generated visual stimuli would then better probe the dependency of two characteristic inherent spatial scales of the biological receptive fields compared to using a single inherent spatial scale for the visual stimuli.

As described in more detail in Lindeberg [16] Section 7, the choice of the angular frequency for sine wave for probing the orientation selectivity properties of visual neurons can significantly affect the shapes of the resulting orientation selectivity curves, and should thus warrant specific consideration.

## Summary and discussion

We have presented a set of three new integrated affine quasi quadrature measures to model the functional properties of complex cells, and analyzed their orientation selectivity properties, based on the assumption that the receptive field shapes should span a variability over the degree of elongation of the simple cells that form the input to these models of complex cells.

This analysis has been performed in three ways; in terms of: (i) orientation selectivity curves, (ii) graphs of the resultant |R| as function of the scale parameter ratio $\kappa =\sigma_{2}/\sigma_{1}$ between the scale parameter $\sigma_{2}$ in the direction perpendicular to the preferred orientation of the receptive field and the scale parameter $\sigma_{1}$ in the direction of the preferred orientation of the receptive field, and (iii) histograms of the resultant |R| when assuming a logarithmic distribution over the scale parameter ratio κ.

Specifically, by qualitative comparisons with the biological results by Nauhaus et al. [22], regarding a significant variability in orientation selectivity properties of biological neurons from wide to narrow orientation selectivity properties, and to Goris et al. [15], regarding orientation selectivity histograms over the resultant |R|, we have found that these results are consistent with a previously proposed hypothesis in Lindeberg [10] further investigated in Lindeberg [14] that the receptive fields in the primary visual cortex should span a significant variability in the degree of elongation of the receptive fields. In this respect, the results are consistent with what becomes a natural consequence of stating desirable properties of an idealized vision system, that the receptive fields should be covariant under the natural geometric image transformations. In such a context, covariance properties of the family of receptive fields enable more accurate estimates of cues to the 3-D structure of the world, as the image data used as input to the vision system undergo significant variabilities, as caused by variations of the viewing conditions, such as the distance and the viewing direction between objects in the world and the observer.

Specifically, by simulating the orientation selectivity histograms that result from the presented new integrated affine quasi quadrature measures ${𝒬}_{\varphi ,12,\text{int}}L$, ${𝒬}_{\varphi ,1234,\text{int}}L$ and ${𝒬}_{\varphi ,34,\text{int}}L$ according to (11), (12) and (13), when combined with an assumption of a uniform distribution over the logarithm of the scale parameter ratio $\kappa =\sigma_{2}/\sigma_{1}$ of the receptive fields, we have found that the extensions of the previous pointwise quasi quadrature measure ${𝒬}_{\varphi ,12,\text{int}}L$ according to (3) offer ways of changing the shapes of the predicted orientation selectivity histograms regarding both how uniform the predicted histograms will be in relation to the previously recorded biological orientation selectivity histograms by Goris et al. [15] and regarding the span of values of the resultant |R| they cover.

Thus, we propose to: (i) include a variability over the degree of elongation of the receptive fields when modelling the computational function of complex cells, (ii) include the mechanisms of spatial integration and including receptive field responses of higher order than 2 when modelling complex cells, and (iii) use similar criteria to match predicted orientation selectivity histograms to biological orientation selectivity histograms, as used in the presented analysis, to evaluate also other types of computational models for complex cells.

Let us finally remark that it should most likely be the case that the non-linear behaviour of complex cells may be more complex than the computational mechanisms used in the proposed idealized models in terms of integrated affine quasi quadrature measures. The overall purpose with this work is on the other hand to demonstrate that the orientation selectivity properties of the proposed idealized models of complex cells can be analyzed with a structurally similar methodology as used for probing the orientation selectivity properties of biological receptive fields. From this viewpoint, the comparison to the biological orientation selectivity histogram accumulated by Goris et al. [15] is specifically to show that the gross behaviour in terms of an underlying distribution of receptive field shapes of different elongation can be used to reflect gross properties of the biological measurements. Our intention in this respect is to stimulate further tests of more complex computational models of complex cells, based on assuming distributions of the underlying receptive field shapes over the degree of elongation. The set of more explicit predictions in the section “Explicit predictions for further modelling of complex cells” are specifically aimed at providing a guide to such further research.

The proposed extension of the methodology for characterizing the orientation selectivity of visual neurons in the section “Explicit suggestion to extension of methodology for experimentally probing the orientation selectivity of complex cells that may comprise a variability in the degree of elongation between different visual neurons”, by instead of performing a one-parameter variation over the orientation of the probing sine wave instead performing a two-parameter variation over both the angular frequency and the orientation of the probing sine wave, is intended to provide richer experimental data that could better distinguish between the explanatory properties of different types of computational models of complex cells.

From the viewpoint of quasi quadrature models to be used for addressing computational tasks in computer vision, it should on the other hand also be mentioned that a hierarchical network constructed by applying a substantially simplified quasi quadrature model of complex cells in cascade can lead to quite reasonable results on computer vision benchmarks (Lindeberg [89]); see also the closely related work by Riesenhuber and Poggio [102], Serre et al. [103] and Pant et al. [104], who construct hierarchical networks for computer vision tasks from sets of idealized models of simple and complex cells coupled in cascade. These studies in computational vision do thus demonstrate that computational mechanisms structurally closely related to the affine quasi quadrature measures proposed and studied in this work can support spatial recognition tasks on visual data.

## Supporting information

S1 File Complementary explanations of main concepts used in the paper Lin25-PONE-suppl.pdf. Explains the terminology regarding “receptive field”, “covariance”, “elongation” and “complex cell”.
(PDF)

S2 FileWolfram Mathematica notebook int-compl-ori-sel.nb.Contains the Wolfram Mathematica commands used for generating the results presented in this paper, and specifically computing (i) the orientation selectivity curves r(φ), (ii) the resultant measures |R(κ)| for different degrees of elongation κ, and (iii) the histograms of the resultant |R| for the idealized models of complex cells proposed in this paper, given logarithmic distributions over the degree of elongation of the models in simple cells used for computing the responses of complex cells by spatially integrated quadratic computations.(NB)
