# Correction: Orientation selectivity properties for integrated affine quasi quadrature models of complex cells

**DOI:** 10.1371/journal.pone.0342936

**Published:** 2026-02-12

**Authors:** Tony Lindeberg

S1 File is broken and cannot be viewed. Please view the correct S1 File below.

## Supporting information

S1 File Complementary explanations of main concepts used in the paper Lin25-PONE-suppl.pdf. Explains the terminology regarding “receptive field”, “covariance”, “elongation” and “complex cell”.
(PDF)

## References

[1] Lindeberg T. Orientation selectivity properties for integrated affine quasi quadrature models of complex cells. PLoS One. 2025;20(9):e0332139. doi: 10.1371/journal.pone.0332139 41021642 PMC12478961

