# Supplementary material for: Correction: Orientation selectivity properties for integrated affine quasi quadrature models of complex cells
Source: PLoS One. 2026 Feb 12;21(2):e0342936. doi: 10.1371/journal.pone.0342936 (PMC12900289; doi:10.1371/journal.pone.0342936)
Supplement: S1 File — Explains the terminology regarding “receptive field”, “covariance”, “elongation” and “complex cell”. (PDF) [file pone.0342936.s001.pdf]

# Supplementary material to “Orientation selectivity properties for integrated affine quasi quadrature models of complex cells”

Tony Lindeberg[1]

**1** Computational Brain Science Lab, Division of Computational Science and Technology, KTH Royal Institute of Technology, SE-100 44 Stockholm, Sweden.
*tony@kth.se

## Abstract

This document contains supplementary material to the paper

> Lindeberg (2025) “Orientation selectivity properties for integrated affine quasi quadrature models of complex cells”, *PLOS ONE.*

regarding explanations of a set of main concepts used in the paper.

## Explanations of main concepts used in the paper

**Receptive field:**

According to the pioneering work by Hubel and Wiesel [1–4], a visual receptive field is defined as the region in visual space that can contribute to the response of a visual neuron. In this work, we follow a functional extension of that approach, by defining a visual receptive field as the computational function that computes the response of a visual neuron to the visual stimuli within the support region of the receptive field, that is over the region in the visual space that can evoke a response of the neuron.

**Covariance:**

The notion of a receptive field being covariant under geometric image transformations means that the receptive field model is well-behaved under the given class of geometric image transformation. If we let $\mathcal{G}$ denote the operator that computes a geometrically transformed image $f'$ from a given input image $f$ according to $f' = \mathcal{G}\, f$, then a receptive field represented by the operator $\mathcal{R}$ is said to be covariant under the corresponding class of geometric image transformations, if the result of applying the receptive field to the geometrically transformed image $\mathcal{R}\,\mathcal{G} f$ can be written as the result of applying a geometric transformation to the result of a related receptive field operator $\mathcal{R}'$ to the same input image according to $\mathcal{R}\,\mathcal{G} f = \mathcal{G}\,\mathcal{R}'\, f$. In this context, the related receptive field operator $\mathcal{R}'$ should either be a different member of the receptive field family defined by the operator $\mathcal{R}$, or constituting a sufficiently simple transformation of a receptive field defined by the operator $\mathcal{R}$.

**Elongation:**

For a visual neuron with a spatial receptive field, one can conceive that the receptive field has different amounts of spatial extent in different spatial orientations in image space. One way to characterize the amount of elongation of a receptive field is by measuring the spatial extent over all possible orientations and then forming the ratio between the extreme values of the spatial extent over all the image orientations. For receptive field models formulated in terms of affine Gaussian derivatives, as used in this work, the receptive field can specifically be decomposed into a smoothing stage with an affine Gaussian kernel followed by spatial derivative computations. For that affine Gaussian derivative model, the degree of elongation $\kappa$ can be defined as the square root of the ratio of the eigenvalues of spatial covariance matrix $\Sigma$ in the affine Gaussian kernel according to Eq. (2) in the main body of the paper as later formalized in Eq. (7) in the main body of the paper.

**Complex cell:**

According to the taxonomy of neurons in the primary visual cortex by Hubel and Wiesel [1–4], a visual neuron is said to be simple if it (i) has distinct excitatory and inhibitory subregions, (ii) obeys roughly linear summation properties and (iii) the excitatory and inhibitory regions balance each other in diffuse lighting. A visual neuron that does not obey these properties is said to be a complex cell. This characterization applies to any single individual visual neuron. In our comparisons to biological data to be performed later in this paper, we will then compare statistics over populations of biological complex cells, for relating gross properties of our proposed computational models to biological data.

# References


1. Hubel DH, Wiesel TN. Receptive fields of single neurones in the cat's striate cortex. J Physiol. 1959; 147:226–238.
2. Hubel DH, Wiesel TN. Receptive fields, binocular interaction and functional architecture in the cat's visual cortex. J Physiol. 1962; 160:106–154.
3. Hubel DH, Wiesel TN. Receptive fields and functional architecture of monkey striate cortex. The Journal of Physiology. 1968; 195(1):215–243.
4. Hubel DH, Wiesel TN. Brain and Visual Perception: The Story of a 25-Year Collaboration. Oxford University Press; 2005.
